# Internal constraints for phenomenal externalists: a structure matching theory

**DOI:** 10.1007/s11229-022-03829-1

**Published:** 2022-08-17

**Authors:** Bryce Dalbey, Bradford Saad

**Affiliations:** 1 University of Texas at Austin, Austin, TX USA; 2 Utrecht University, Utrecht, Netherlands; 3 Sentience Institute, New York, USA

**Keywords:** Intentionalism, Naturalism, Phenomenal externalism, Phenomenal structure, Tracking intentionalism, Perceptual variation, Missing shades

## Abstract

We motivate five constraints on theorizing about sensory experience. We then propose a novel form of naturalistic intentionalism that succeeds where other theories fail by satisfying all of these constraints. On the proposed theory, which we call *structure matching tracking intentionalism*, brains states track determinables. Internal structural features of those states select determinates of those determinables for presentation in experience. We argue that this theory is distinctively well-positioned to both explain internal-phenomenal structural correlations and accord external features a role in fixing phenomenology. In addition, we use the theory to shed light on how one comes to experience “missing shades”.

## Introduction

This paper develops a theory of what sorts of non-mental features fix sensory phenomenology, i.e. the character of subjects’ sensory experiences. We develop the theory within the frameworks provided by two research programs: the naturalization program of ultimately explaining the mind in non-mental terms and the intentionalist program of understanding experience in terms of representation. Like existing naturalistic intentionalist theories, the theory we propose is programmatic. But it goes beyond existing naturalistic intentionalist theories of experience by solving outstanding problems for them.

To set the stage, in Sect. 2 we revisit a constellation of veridical, hallucinatory, and brain-in-vat cases and use verdicts about them to generate a set of constraints on theorizing about experience. These constraints turn out to motivate a view on which certain external features help fix content and in turn phenomenal character. In particular, they motivate what we’ll call *simple tracking theories* on which (very roughly) what a subject experiences is fixed by what her brain states track. However, in Sect. 3 we use empirical considerations to argue against these theories. Specifically, we use observations about the structure of subjects’ cognitive, behavioral, and neural profiles to support two additional constraints. We then show that simple tracking theories violate these constraints. While this conflict could be taken as a basis for rejecting naturalistic intentionalism, we instead propose a more sophisticated naturalistic intentionalist theory in Sect. 4. This theory, which we call *structure matching tracking intentionalism*, mirrors some recent proposals in the psychosemantics literature in according a substantive content-fixing role not only to relations between internal states and environmental features but also to the structure of internal states.^1^ On the resulting picture, brain states track determinable qualities. Internal structural features of those states then select determinates of those determinables for presentation in experience. After showing how structure matching tracking intentionalism satisfies the noted constraints, we conclude by illustrating a further virtue of the view: its ability to solve the problem of missing shades, roughly the problem of accounting for experiences of sensory qualities absent from the environment.

## A path to simple tracking theories

Experiences are states that there’s something it’s like to undergo.^2^ What it is like for a subject to undergo an experience is the phenomenal character of the experience. When a subject has an experience that presents her with *F*—i.e. which makes it phenomenally seem to her that *F* is instantiated—we can say that she has an experience as of *F*. This is not to say or imply that *F* is instantiated. For instance, it is coherent to claim both that someone has a hallucinatory experience as of blueness and that blueness is uninstantiated.

In what follows, we are only concerned with sensory experience. We assume that sensory experiences are as of ostensibly external properties. For instance, when you see a traffic light change, your visual experience presents you with temporal and color properties, properties which phenomenally seem to you to belong to an object in your environment. Call properties that can be presented in sensory experience *sensory qualities*. As we will see, there are different views about the nature of sensory qualities. For brevity, we often implicitly relativize ‘experience’ and related expressions to the sensory.

What non-mental features fix the phenomenal character of (sensory) experience? We understand ‘fix’ in terms of nomic necessitation: *x* fixes *y* just in case *x* explains and (at least) nomically necessitates *y*. It is the business of naturalistic theories of experience to answer this question. On this broad construal, naturalistic theories encompass forms of physicalism, functionalism, Russellian monism, and dualism.^3^ There is room for disagreement among such theories about how non-mental features explain phenomenal character. For instance, whereas physicalists will hold that non-mental features constitutively explain phenomenal character, dualists may deny this, maintaining instead that non-mental features merely causally explain phenomenal character.^4^

In theorizing about what fixes phenomenal character, it's reasonable to begin by considering ordinary veridical experience. Suppose Ruth is a human who sees a blueberry in good lighting. She has a veridical perceptual experience as of blueness and roundness.^5^ In this case, the character of her experience systematically depends on how she is related to sensory qualities: had she perceived a chocolate bar instead of a blueberry, she would have experienced brownness and rectangularity instead of blueness and roundness. This fact suggests that theories of experience should respect:

**Relationality**: How one is related to sensory qualities helps fix phenomenal character.

Adopting Relationality as a constraint, we may seek a theory of experience that respects it. To respect it, a theory must by and large yield the wanted verdicts in the range of cases that motivate Relationality. The same goes for respecting other constraints that we will consider. A salient and extremely simple theory that respects Relationality—which we’ll call the *perceptual theory*—holds that perception of sensory qualities fixes phenomenal character. On this view, *S* has an experience as of *F* iff *S* perceives *F*.^6^

However, other cases reveal that the perceptual theory is incorrect. Suppose Ruth hallucinates a blueberry. She has an experience as of blueness and roundness even though nothing before her is blue or round. Or suppose that Ruth hallucinates a chocolate bar. She has an experience as of brownness and rectangularity even though nothing before her is brown or rectangular. The differences in which sensory qualities her experiences present are not explained by differences in which sensory qualities she perceives, as she does not perceive the relevant qualities. Such hallucinatory cases also show that whatever fixes phenomenal character does not require experienced sensory qualities to be present before one. This suggests that whatever fixes phenomenal character is representational—as representation imposes no such requirement—and hence that theories of experience should respect:

**Intentionality**: Which sensory qualities one represents helps fix phenomenal character.^7^

Perhaps the most straightforward way to respect Relationality and Intentionality is to opt for *intentionalism* on which the qualities presented in experience are fixed by the subject’s standing in a representation relation to those qualities. Naturalistic versions of intentionalism require that the relevant form of representation ultimately be explained in non-mental terms. For those of us in the market for such a theory, the vast external physical differences between subjects of veridical and hallucinatory experiences provides an initial reason to embrace *internalism*, the thesis that the physical basis of phenomenology is internal (i.e. is fixed by the intrinsic features of the subject). The result is *(naturalistic) internalist intentionalism*, on which there is a naturalistic representation relation *R* such that internal physical features fix what one bears *R* to and *S* has an experience as of *F* iff *S* bears *R* to *F*.

While internalist intentionalism tells us that there is some such relation *R*, it does not independently specify that relation. But even in advance of such specification, another sort of case spells trouble for internalist intentionalism. Suppose that, in Swampman fashion, an envatted cantaloupe-sized lump of gray matter—*Swampy*—materializes in a distant galaxy as the result of an unlikely but nomically possible quantum fluctuation.^8^ It happens to be envatted in a manner that places it in the same internal physical state as Ruth’s brain when Ruth veridically experiences a blueberry. Swampy is physically isolated from blueness and roundness to the extent allowable by the laws of nature. It does not perceive any round objects and nothing in its environment is blue. Having just come into existence and not being a member of a species, it does not stand in any significant historical relations to blueness or roundness. Being both forever unembodied (unlike the original Swampman) and able to be embodied in many ways, it is not the case that Swampy would behave in blue-appropriate or round-appropriate ways were it embodied. Given these stipulations, what, if anything, is it like to be Swampy?

On internalist intentionalism, what it is like to be Swampy is the same as what it is like to be Ruth when she veridically experiences a blueberry. Hence, given that Ruth has an experience as of blueness and roundness, internalist intentionalism predicts that Swampy does too. Is this answer correct? Well, the stipulations of the case do not a priori entail this answer or, for that matter, any answer to this question. (We will revisit this issue in Sect. 3.) But we can bring some considerations to bear. Given the dearth of significant, discriminating physical relations Swampy bears to blueness and roundness, Swampy plausibly does not have an experience as of blueness and roundness.^9^ Since internalist intentionalism predicts that Swampy has such an experience, internalist intentionalism is implausible. In fact, this sort of reasoning applies to a broad range of isolation cases—cases containing objects that are internal physical duplicates of conscious subjects but which are stripped of significant physical connections with sensory qualities.^10^

To avoid implausible predictions about isolation cases, internalist intentionalists could identify sensory qualities with certain internal physical states. For example, they could counterintuitively claim that roundness is a certain neural state of Ruth and Swampy rather than a property of blueberries. Or, they could allow that roundness is a property of blueberries rather than a neural state but insist that Swampy’s case implicitly features a suitable internal physical connection to that property, the apparent dearth of such connections notwithstanding. Yet another option for internalists: sever the predictive link between what Swampy experiences and what sensory qualities it is physically isolated from by denying that experiences present subjects with sensory qualities.^11^ For those of us who find such internalist responses difficult to embrace, an alternative moral suggests itself, namely that merely varying external (i.e. purely environmental) features can induce phenomenal variation and hence that theories of experience should respect:

**Externalism**: External features help fix phenomenal character.

Theories that respect this constraint lend to a simple explanation of why Swampy doesn’t experience blueness and roundness: due to its isolation, Swampy fails to enjoy a suitable physical connection to blueness and roundness.

It may seem that hallucinatory cases are less fanciful isolation cases. After all, in both, sensory qualities are absent from the immediate environment. Consequently, it may seem that no theory that respects Externalism will also successfully generate different verdicts in the two sorts of case. Fortunately, certain externalist intentionalist theories have been proposed that solve this problem. On those theories—which we'll call *simple tracking theories*—internal physical duplicates can phenomenally differ due to differences in what they “track”.^12^

Tracking is a naturalistic representation relation that reaches outside the head. It is partly analyzable in terms of optimal conditions and a response relation: a state tracks a property if the state would, when in optimal conditions, suitably respond to that property. But a state can still track a property when it does not respond to the property, provided that the state would suitably respond to that property if it were in optimal conditions. Therein lies the possibility of misrepresentation.

By according tracking a role in fixing phenomenology, simple tracking theories reject internalism. Nevertheless, they invoke internal features: on these theories, it is because internal physical states occupy a certain internal and naturalistic functional role that they are eligible to give rise to experience. We will call this the *e-role* (for ‘experience-role’).^13^ Still, these theories do not appeal to internal features to explain phenomenal differences between experiences—they explain phenomenal differences in terms of differences in which external features are tracked.^14^ This is not to say that these theories are strictly inconsistent with the existence of such constraints: if a simple tracking theory is correct and tracking external features is itself substantively constrained by internal features, phenomenology may be substantively constrained by internal features. We will revisit this possibility in Sect. 3.3.

We can arrive at an illuminating taxonomy of simple tracking theories by distinguishing four dimensions along which they vary. First, they differ on what they take responding to a property to be. Some take it to be a matter of (causally, explanatorily, or modally) covarying with its presence.^15^ Others take it to be a matter of standing in an indication relation to it.^16^ There are also output-oriented accounts, e.g. on which responding to a property is a matter of exhibiting a fitness-enhancing behavioral profile because of the presence of that property.^17^ Another option—and one that has received surprisingly little discussion—is that responding to a property is a matter of bearing a perceptual relation to it.^18^ Further candidates can be generated by imposing a restriction, e.g. an asymmetric dependence requirement, on the foregoing relations.^19^

Second, simple tracking theories also vary regarding what the relevant notion of optimal conditions is. One option is to construe optimal conditions for a state as those in which it (or a feature of it, such as responding to a property) was selected for.^20^ Others appeal to conditions in nearby worlds in which responding to a property contributes to the inclusive fitness of the organism that bears the state.^21^ Still others appeal to the historically dominant conditions in which the state was instantiated.^22^ Further options appeal to conditions under which interfering features are absent.^23^ On some of these, what conditions count as optimal for a state are determined by the state’s history, while on others it is fixed by the state’s forward-looking profile. In what follows, we remain neutral on how optimal conditions are to be construed.

Third, while simple tracking theories agree that a functional role renders internal physical states eligible to give rise to experience and, in addition, that it is naturalistic and internal, they disagree on exactly what the relevant functional role is. The literature contains a host of candidates for the e-role, each of which can be incorporated into a simple tracking theory. These candidates include being poised for cognitive access, being accessible to the global workspace, being the object of a certain sort of higher-order mental state, maximizing the quantity of integrated information, and being engaged in recurrent processing.^24^

A fourth dimension of variation in the space of simple tracking theories concerns the ontological status of tracked features. Take the blueness Ruth experiences when she sees the blueberry. On some simple tracking theories, that color quality is a physically reducible quality, perhaps a surface reflectance profile.^25^ On others it is a primitive quality.^26^ On still others it is a quiddity-involving quality.^27^ It is common ground among simple tracking theories, as we’ll understand them, that sensory qualities are response-independent. We'll assume this as well while remaining neutral throughout between different response-independent accounts of sensory qualities.^28^

Officially, then, simple tracking theories add to externalist intentionalism that *S* has an experience as of *F* iff *S* is in an e-role occupying internal physical state that tracks *F*. Having introduced them, we can see how simple tracking theories manage to respect Externalism while generating the correct verdicts about both hallucinatory and isolation cases. Per their appeal to tracking, such theories respect Externalism. Now recall Ruth when she hallucinates a blueberry even though nothing before her is blue or round. In this case, Ruth has an experience as of those qualities. But she is also isolated from them insofar as they are absent from her immediate environment. However, this is a rather limited sort of isolation. Witness the many physical connections between Ruth and blueness as well as roundness. For instance, this sort of isolation and the stipulations of Ruth’s hallucinatory case are compatible with her being in a(n e-role occupying internal physical) state that, absent interference, covaries with blueness and roundness.^29^ And they are compatible with her being in a state that was selected for in Ruth’s species because it causally covaried with blueness and roundness. Thus, provided that simple tracking theories identify tracking with a physical relation that reaches beyond subjects’ immediate environments, these theories have no difficulty in accounting for cases like Ruth’s in which hallucinating subjects have experiences as of qualities despite those qualities not being present before them.

Accommodating hallucinatory cases in this manner does not get simple tracking theories in trouble with isolation cases like Swampy’s. Swampy is not just locally isolated from blueness and roundness. It is not connected to blueness or roundness via any of these candidate tracking relations since, for example, it lacks a personal history and does not belong to a species with an evolutionary history. In fact, as we have seen, Swampy plausibly does not bear any significant, discriminating physical relation to those qualities. As a result, unlike hallucinating Ruth, Swampy is not in a state that tracks blueness and roundness. It is for this reason that simple tracking theories can accommodate hallucinatory cases while avoiding the implausible prediction that Swampy has an experience as of those qualities. Ordinary veridical cases also do not pose a problem for these theories: on them, when Ruth sees the blueberry and has an experience as of blueness and roundness, she has that experience because she is in a state that tracks those qualities. (Indeed, it is natural for simple tracking theorists to take many cases of veridical experience to be cases in which optimal conditions obtain while a state responds to a quality.)

In addition to handling a broad range of cases and respecting Externalism, simple tracking theories also straightforwardly respect Relationality and Intentionality. They respect Relationality by holding that which sensory qualities one’s states track fixes phenomenal character. And they respect Intentionality since they appeal to tracking and tracking is a representation relation. The moral we draw from these reflections is that, while there is much controversy about how the details of simple tracking theories should go, they advocate an especially promising naturalistic picture of how phenomenal character is fixed.

## Structural problems for simple tracking theories

We have seen that simple tracking theories exhibit distinctive promise. But some have rejected them—and, indeed, all forms of externalism about experience—on the basis of certain armchair intuitions. According to these intuitions, certain internal physical duplicates—for instance, a Boltzmann brain which pops into existence for ten seconds, during which it is an internal physical duplicate of Ruth's brain—share phenomenology.^30^ Externalist intentionalists have for the most part conceded that their view incurs significant costs in running afoul of these intuitions while maintaining that these costs are worth paying.^31^

In our view, this is a mistake. To see why, consider the following trilemma.^32^ Such intuitions either originate from a priori insight or have an a posteriori source, either by tracing to common sense or to more sophisticated empirical considerations. It is implausible that they are the deliverances of a priori insight since, as reflection on zombies shows, there is no clear a priori entailment from physical facts to facts about experience. It is also implausible that the intuitions express the common sense view: if anything, common sense favors naive realist forms of externalism on which features of a subject's environment constitute their experience. Lastly, if such intuitions find their source in more sophisticated empirical considerations, the intuitions should be explicated in terms of those considerations. As far as we know, this has not been done, though some philosophers have challenged externalism by using detailed empirical considerations rather than armchair internalist intuitions. We repurpose some of these considerations below to motivate fruitful constraints on an externalist view of experience.

In light of the above trilemma, simple tracking theories should not be rejected on the basis of armchair intuitions. Nonetheless, some scenarios—including some that have been used to elicit those intuitions—can, with the help of empirical considerations, be brought to bear on externalist intentionalist theories. These include Swampman, Inverted Earth, Swampman Goes to Inverted Earth, Russell World, El Greco World, Temporal El Greco World, Doubled Earth, Mabel and Maxwell, Middle Earth Mary, Killer Yellow, and Missing Shade of Blue.^33^ On the surface, these problem scenarios constitute a heterogeneous lot. But, on reflection, they all pose problems for simple tracking theories that have a common form: the problems arise because the scenarios seem to dissociate relevant internal structures from the structure of tracked features and thereby elicit implausible predictions from simple tracking theories about the relationship between phenomenal structure and the structure of cognition and behavior.^34^ For instance, these theories predict that in a wide range of such scenarios the unitary-binary structure of color experience comes apart from the unitary-binary structure of color cognition and behavior. As we will see, empirical considerations tell against such systematic divergence.

Contrast cases provide a clear way to see how such scenarios pose a problem for simple tracking theories. Some contrast cases pose problems for these theories by holding constant internal features while varying certain external structural features—call these *SIDE cases* (for same internal, different external). Others pose problems by varying internal structural features while holding constant certain external features—call these *DISE cases*. The SIDE-DISE distinction marks a rarely appreciated theoretical-joint among problems for these theories. The task of this section is to spell out how each sort of case poses a problem for simple tracking theories and yields a distinct constraint on what fixes phenomenal character. One constraint imposes a form of internal sensitivity on phenomenal character while the other imposes a form of external insensitivity. An adequate successor theory should respect both of these constraints, along with Relationality, Intentionality, and Externalism. Section 4 proposes such a theory.

### The Mabel and Maxwell Scenario

This subsection presents a single pair of SIDE and DISE cases. They constitute a variant of Pautz’s Mabel and Maxwell Scenario.^35^ We will initially describe Mabel and Maxwell abstractly to highlight crucial aspects of the Scenario. We will then, for concreteness, fill the Scenario out using color and color experience.^36^ After that, we elicit predictions from simple tracking theories about Mabel’s experience. In Sect. 3.2, we will see that these predictions are incorrect.

To start, meet our control subject, Maxwell. He is a normal human residing in a nomically possible world. One day, Maxwell encounters a stimulus. Upon encountering the stimulus, Maxwell experiences a quality of the stimulus. The structure of his experience reflects structure of the experienced quality. Likewise, Maxwell’s behavior and cognition reflect the structure of his experience, and hence the noted structure of the quality. Moreover, a neural state underlies that experience and tracks the relevant features of the stimulus. For concreteness, let’s say that Maxwell sees an orange paint chip. Upon viewing the paint chip, he experiences orange. His experience exhibits binary structure that reflects the binary structure of orange.^37^ Likewise, he systematically exhibits binary color cognition and behavior, e.g. he judges and reports that he is having an experience of a paint chip with a color that is a mixture of two other colors. Call the neural state underlying Maxwell’s binary color experience *N*_*B*_. N_B_ occupies the e-role and tracks some property P. Let *Binary Maxwell* denote Maxwell in this case.

On another day, Maxwell is exposed to a stimulus with a structurally different quality. Upon encountering the stimulus, Maxwell experiences that quality. On this occasion, his experience reflects the structure of that quality that distinguishes it from the quality Maxwell experienced on the first occasion. But, as on the first occasion, Maxwell’s behavior and cognition here reflect the structure of his experience, and hence the noted structure of the quality. As before, an e-role occupying neural state underlies his experience and tracks the relevant features of the stimulus. For concreteness, let’s say that Maxwell sees a red paint chip. Upon viewing the paint chip, he experiences red. His experience exhibits unitary structure that reflects the unitary structure of red. Likewise, he systematically exhibits unitary color cognition and behavior. Call the neural state underlying Maxwell’s unitary experience *N*_*U*_. N_U_ occupies the e-role and tracks some property P*.^38^ Let *Unitary Maxwell* denote Maxwell in this case. Binary Maxwell’s and Unitary Maxwell’s local environments are as similar as possible given the foregoing stipulations. In neither case are Maxwell’s circumstances exceptional: Maxwell is not subject to relevant psychiatric disorders, perverse incentives, or factors that interfere with how he reacts to the stimulus.

The stipulation that Binary Maxwell and Unitary Maxwell respectively have binary and unitary color experiences requires clarification. We can get a grip on such experiences by example: binary color experiences include experiences as of orange and experiences as of purple; unitary color experiences include experiences as of red, yellow, and blue. There is a salient kind of structure that is aptly called ‘binary’ presented by the phenomenal character of the former experiences. Likewise, there is a salient kind of structure that is aptly called ‘unitary’ presented by the phenomenal character of the latter experiences.^39^ On simple tracking theories, for Binary Maxwell and Unitary Maxwell to have experiences that present these structures, Binary Maxwell must be in a state that tracks binary structure while Unitary Maxwell must be in a state that tracks unitary structure. Thus, the properties they track—namely P and P*—must reflect these structures.

One might worry that, given a standard physicalist account of sensory qualities, such structural features are not tracked, as, for instance, there is nothing binary or unitary about reflectance profiles. Two responses are available for simple tracking theorists. First, they may recover the relevant structural features using resources available to physicalist accounts of sensory qualities. Second, they may eschew standard physicalist accounts of sensory qualities in favor of a primitivist or quiddity-involving account that more readily accommodates such structural features.^40^

Next, meet our test subject, Mabel. She inhabits the same world as Maxwell. She is a normal member of a different human-like species that evolved the same receptor system as humans, but different post-receptoral wiring. In particular, her post-receptoral wiring ensures that exposing her to the stimulus Binary Maxwell encountered (in a near-duplicate local environment) prompts her to enter Unitary Maxwell’s neural state. In Mabel, that neural state tracks whatever Binary Maxwell’s neural state tracks. As a result, the structure that her neural state tracks diverges from the structure of her associated cognition and behavior. Thus, upon looking at the orange paint chip, Mabel enters N_U_, which occupies the e-role and tracks P.^41^ And she (like Unitary Maxwell) systematically exhibits unitary color cognition and unitary behavior.^42^ Her circumstances are not exceptional: she is not subject to any relevant psychiatric conditions, perverse incentives, or interfering factors. Note that facts about Mabel’s experience after she enters N_U_ have not been stipulated.

We can depict these subjects as follows:
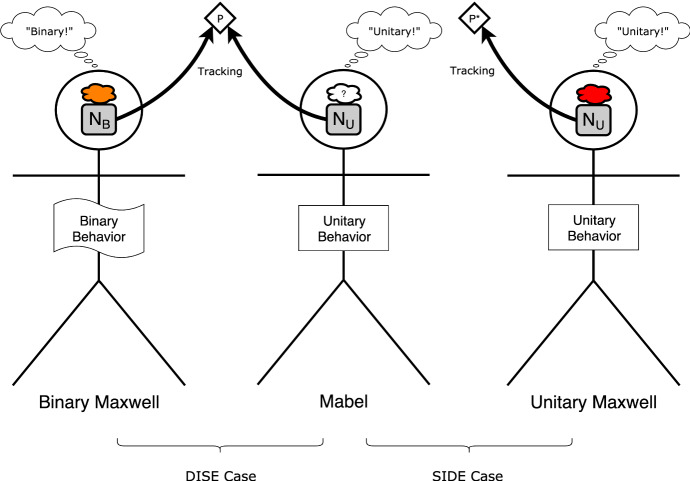


Taken with Binary Maxwell, Mabel yields a DISE case: while Mabel and Binary Maxwell exhibit internal structural variation, their relevant internal states track the same external features. Taken with Unitary Maxwell, Mabel yields a SIDE case: while Mabel and Unitary Maxwell are internally alike, they are in internal states that track different structural features.

Simple tracking theories make predictions about both cases. They predict that Mabel and Binary Maxwell have identical color phenomenology, as their neural states track the same feature (namely P). They also predict that Mabel and Unitary Maxwell have structurally distinct color phenomenology, as their neural states track structurally distinct features (P and P*). Are these predictions correct? We will now provide reasons to think that they are not.

### Structural problems and constraints

We will now argue that the Mabel-Maxwell SIDE and DISE cases pose problems for simple tracking theories and yield constraints on theories of experience. Let’s start with the DISE case.

Recall that Binary Maxwell exhibits binary color cognition and behavior while Mabel exhibits unitary color cognition and behavior. Now, in actual humans the structure of color phenomenology is reflected in their accompanying cognition and behavior. For instance, subjects sort color chips into hue classes in a way that systematically classifies certain chips as being of only one hue.^43^ This structural match suggests that there is an explanatory connection between the structure of color phenomenology and the cognition and behavior that reflect it. Given such a connection, we should expect that subjects who exhibit systematic differences in the structure of their color cognition and behavior will, all else equal, exhibit at least some differences in the structure of their color phenomenology. By construction, Mabel is not exceptional in ways that exempt her from this regularity. These considerations give us reason to think that Mabel and Binary Maxwell have structurally different color phenomenology.

Here is a second, related reason to think that Mabel and Binary Maxwell exhibit structurally different color phenomenology. According to prominent neuroscientific theories, the unitary/binary structure of color experience is also reflected in the neural processing underlying vision.^44^ Provided that those theories are on the right track, N_B_ and N_U_ exhibit binary-unitary structure that covaries with binary-unitary color experience in actual humans. We should thus expect that subjects in neural states which differ with respect to unitary-binary structure of color processing will, all else equal, exhibit at least some differences in the structure of their color phenomenology. Granted, this connection may be severed under exceptional circumstances. But again, Mabel's circumstances are not exceptional. These considerations also give us reason to think that Mabel and Binary Maxwell have structurally different color phenomenology.

We saw above that simple tracking theories instead predict that Mabel and Binary Maxwell undergo structurally identical experiences. This case reveals a general difficulty for these theories: they err in allowing phenomenal structure to float free from neural, cognitive, and behavioral structure. This suggests the following as an additional constraint that an adequate theory of experience should meet:

**Internal structural sensitivity**: Internal structural features help fix phenomenal structure.

Here, internal structural variation is to be understood in a broad manner that encompasses structural variations in intrinsically determined cognitive and behavioral profiles, neural structural variations, and combinations of these variations. To yield the correct verdicts in DISE cases, a theory will need to (directly or indirectly) appeal to internal features that have both cognitive and behavioral structural import. Rather than appealing directly to internal cognitive and behavioral structure, a theory might appeal to underlying neural structure. It is an interesting question what sorts of internal structure a theory should render phenomenal structure sensitive to in the first instance. But a resolution to this issue is not suggested by reflection on cases like Mabel and Maxwell’s. We remain neutral on it in what follows.

Internal Structural Sensitivity and Externalism, in concert with Relationality and Intentionality, call for an intentionalist view on which both internal and external features substantively constrain phenomenology. In short, they call for a *two-factor* form of intentionalism.

Let’s return to the SIDE case. Recall that Mabel and Unitary Maxwell exhibit unitary color cognition and behavior while undergoing N_U_. As noted above, in actual subjects the structure of color phenomenology is reflected in their accompanying behavior, cognition, and neural processing. This systematic structural match suggests that there is an underlying explanatory connection. Given such a connection, we should expect that subjects who undergo similar neural processing and exhibit structurally similar color cognition and behavior will, all else equal, exhibit similar structure in their color experiences. Granted, all else may not be equal if the subject suffers from some psychiatric condition, falls prey to interfering factors, or has been incentivized to systematically change their color cognition and behavior. But, by stipulation, Mabel is not exceptional in these ways. These considerations support the conclusion that Mabel’s color phenomenology is structurally similar to Unitary Maxwell’s. Simple tracking theories run afoul of this result: as we have seen, they predict that Mabel has binary color phenomenology while Unitary Maxwell has unitary color phenomenology.

As this SIDE case illustrates, we should expect phenomenal structure to remain invariant under alterations in tracked structure, provided that internal structure is held constant. That is:

**External structural insensitivity**: Certain external structural features do not help fix phenomenal character.

This constraint captures the form of insensitivity that is demanded by Mabel-Maxwell SIDE cases. The external structural features that fall within its scope are precisely those in Mabel-Maxwell SIDE cases that lead simple tracking theories to make predictions about phenomenal structure which run contrary to what is suggested by the cognitive, behavioral, and neural profiles of the subjects. In the described color Mabel-Maxwell SIDE case, this is the binary-unitary structure exhibited by the paint chips in their environments. External Structural Insensitivity entails analogous conclusions in cases involving other sense modalities. Given Externalism and External Structural Insensitivity, phenomenology is sensitive to only some external features. For reasons given in Sect. 3.3, exactly which features is a difficult matter and not something we will try to resolve.

### The guaranteed match objection

There is an instructive objection that simple tracking theories might appeal to in order to resist the charge that they yield incorrect predictions about the Mabel-Maxwell Scenario. The objection starts with the observation that the stipulations of the Scenario require the nomic possibility of a structural mismatch between a quality and a state of Mabel that tracks that quality. However, according to the objection, every quality (that is a candidate for P) is *match guaranteeing*, i.e. such that it is nomically necessary that any e-role occupying internal physical state that tracks the quality also matches it in structure.^45^ So, the Scenario is nomically impossible, and hence not a source of counterexamples to simple tracking theories.

Admittedly, some candidates—for instance, shapes—may be match guaranteeing in this way. To see this, consider a version of the Scenario on which, when Mabel and Maxwell are in the same neural state, they respectively track roundness and triangularity. As stipulated, both subjects are in unexceptional circumstances (e.g. are not fitted with shape-permuting goggles) and systematically exhibit triangle-appropriate behavior. This version may well be nomically impossible. For instance, suppose optimal conditions are those under which a state’s response to a property was selected for. Then neural states that respond to roundness while inducing triangle-appropriate behavior and cognition might be necessarily ineligible to track roundness as a result of that response being necessarily maladaptive. Plausibly, human-like creatures that failed to exhibit such behavior would have difficulty surviving long enough to pass along their genes. If so, Mabel’s neural state will not track roundness while respecting the laws of nature that hold in our world. Since the argument requires that the Scenario is nomically possible, this version of the argument will then fail.

However, even if shapes guarantee internal-tracking matches, many secondary qualities (colors, sounds, tastes, etc.) do not: many such qualities permit internal-tracking mismatches of the sort Mabel exhibits. Admittedly, simple tracking theorists might try to show in any of several ways that all qualities are match guaranteeing: they could appeal to features of states that are eligible to track qualities, features of the qualities that are eligible to be tracked, or features of the tracking relation. Of these, the only initially promising way is to argue that some features of the tracking relation preclude internal-tracked structural mismatches. But it is very hard to see how this strategy might be implemented. The trouble is that nothing about any of the tracking relations on offer enforces a general ban on internal-tracking structural mismatches.

Indeed, on virtually any of the going accounts of optimal conditions and response relations, it is nomically possible for a state in optimal conditions to respond to (say) a color quality and yet fail to match it in structure. Since responding to a quality in optimal conditions is sufficient for tracking a quality, it follows that the relevant sort of internal-tracking mismatch is nomically possible on such accounts.

To illustrate, consider a no-interference account on which optimality is a matter of interfering factors being absent. In ordinary viewing conditions actual subjects exhibit widespread disagreement—in ways that systematically vary, for example, with age, race, and sex—about whether a given paint chip is true blue (i.e. blue not tinged with any other colors) rather than, say, a particular shade of greenish-blue. Plausibly, in the absence of any sort of interference a quality of the chip elicits unitary responses from some subjects and binary responses from others. If so, then on the no-interference account, these structurally different states track the same quality. Thus, there is reason to think that actual scenarios in which subjects disagree about whether a stimulus is true blue feature the requisite sort of mismatch.^46^

The foregoing objection fares no better if optimal conditions are construed evolutionarily as those in which a state was selected to respond to a given property. Here, the trouble is that, plausibly, it’s nomically possible for a state’s responding to (say) a shade of greenish-blue to be selected for even if the structure of the state diverges from the structure of that color.

For instance, it could be that a human-like species inhabits an environment in which prevalent nutritious and poisonous fruits have distinct but very similar shades of greenish-blue and members of that species undergo states that exhibit very different structures (e.g. unitary and binary) while responding to these very similar colors. This allows those creatures to more easily distinguish the nutritious from poisonous fruits than the other members of the species (who, say, undergo states with very similar structure that match the shades). There would then be selection pressures toward a state’s responding to a shade of greenish-blue when the state’s structure does not match that color’s structure. Admittedly, such selection pressures do not ensure that such a mismatching response is selected for: such pressures might be outweighed by countervailing selection pressures that favor matching responses to that color. But for qualities like greenish-blue such countervailing selection pressures could very well be absent.^47^ So, plausibly, a structural mismatch between a shade of greenish-blue and a state is compossible with the state’s response to that color being selected for.

Additionally, on natural ways of filling in the details of the foregoing scenario, the presence of a shade of greenish-blue will be the dominant historical source of a state that does not match it in structure. Thus, accounts that equate optimal conditions with historically dominant conditions also permit the requisite sort of mismatch.

So, the guaranteed match objection fails as a general strategy for shielding simple tracking theories from incorrect predictions about the Mabel-Maxwell Scenario. Nonetheless, the objection is instructive, as it sheds light on External Structural Insensitivity. As noted in Sect. 3.2, which external features fall within the scope of that constraint is a difficult matter. One reason this is a difficult matter is that match guaranteeing qualities lie outside the scope of the constraint and, while there is reason to think that some qualities are match guaranteeing while others are not, which qualities guarantee internal-tracking matches is also a difficult matter.

## Summary: the problem of structural correlations

To sum up, Sect. 3.1 introduced the Mabel-Maxwell Scenario, used it to generate DISE and SIDE cases, and elicited predictions from simple tracking theories about what experiences Mabel undergoes in those cases. Section 3.2 argued that simple tracking theories’ predictions about those cases are incorrect and gleaned two constraints from those cases, namely Internal Structural Sensitivity and External Structural Insensitivity. Section 3.3 raised and answered the objection that the Scenario is nomically impossible because qualities guarantee internal-tracking structural matches.

The core of the foregoing argument can be understood in terms of what we’ll call the *problem of structural correlations*. The problem arises from the fact that, while there are systematic correlations between the structure of phenomenology and the structure of internal states, simple tracking theories explain phenomenal structure in terms of tracked structure. Although we have focused on unitary-binary structure in the Mabel-Maxwell case, various sorts of phenomenal structures can generate the problem by corresponding to internal physical structure but not to tracked structure. These include other compositional structures, as well as phenomenal structures consisting of magnitudes,^48^ determinate-determinable relations, exclusion relations,^49^ and resemblance.^50^ At least some of these internal-phenomenal structural correlations are striking. However, because simple tracking theories tether phenomenal structure to tracked structure, they have difficulty accounting for these correlations.

Simple tracking theories may be able to explain why there is an internal-phenomenal-tracked structural match in the case of shapes. But what may go for shapes does not go for all sensory qualities. The trouble is that none of the accounts of tracking on offer provide any reason to think that internal structure is generally beholden to tracked structure. Colors may not be the only qualities that give rise to the problem of structural correlations: there is reason to think that qualities like taste intensity, pitch, and pain generate the problem as well. In any case, simple tracking theories’ inability to explain the structural correlations for color experience is problem enough.

## Structure matching tracking intentionalism

We argued in Sect. 3 that while simple tracking theories succeed where previously considered theories fail by respecting Relationality, Intentionality, and Externalism, they are not without fault: they violate Internal Structural Sensitivity and External Structural Insensitivity. Granted, if these theories were the only contenders for satisfying Relationality, Intentionality, and Externalism, we would have reason to revisit that argument. However, there is another contender that satisfies these three constraints as well as Internal Structural Sensitivity and External Structural Insensitivity.

Enter: structure matching tracking intentionalism. Like simple tracking theories, this theory is a naturalistic form of intentionalism. It also follows simple tracking theories in appealing to tracking and the e-role. However, on structure matching tracking intentionalism, what e-role occupying states track does not by itself fix phenomenology. Internal structure of such states also plays a role. The picture is one on which brains access sensory qualities by tracking determinables (e.g. having color) in their environment. Internal structure then selects determinates of those determinables for presentation in experience. The theory can be stated more formally as follows.

*Structure matching tracking intentionalism*: *S* has an experience as of *F* iff *S* is in an e-role occupying internal physical state *x* such that:

1. *x* tracks a determinable *D* which has *F* as a determinate, and
2. *F* is the determinate of *D* whose structure most closely matches *x*'s internal structure.

To illustrate, suppose that Ruth has an experience as of a certain shade of blue, say, true blue. Given structure matching tracking intentionalism, Ruth will then be in a(n e-role occupying internal physical) state that tracks a determinable of true blue. Further, true blue will be the determinate of that determinable that Ruth’s state most closely matches in structure. In nearby worlds in which her state tracks the same determinable but has a structure that most closely matches the structure of a different determinate of it—say, a shade of greenish-blue—Ruth will instead have an experience as of that determinate. Similarly, holding constant the structure of Ruth’s state while varying which determinable that state tracks may result in the structure of that state most closely matching the structure of a different determinate of the tracked determinable; if so, then which determinate Ruth has an experience as of will correspondingly vary. For instance, in such scenarios, rather than having an experience as of true blue, Ruth might have an experience as of a non-blue color in our quality space, a color quality outside of our color quality space, or a non-color quality.^51^

Some remarks will serve to further elucidate structure matching tracking intentionalism and highlight some questions the theory raises but does not answer. *First*, we have left the operative notion of structure at an intuitive level. By precisifying this notion in different ways, we can generate different precisifications of structure matching tracking intentionalism. The same goes for the operative notion of matching. Some precisifications are obvious non-starters: for example if the adopted notions of structure and structure matching jointly make structure matching too easy, then virtually every internal physical state’s structure will perfectly match that of virtually every sensory quality’s, in which case the resulting theory will not be able to exploit structure matching to explain why experience presents us with certain determinates of a tracked determinable but not others.^52^

One approach to avoiding this pitfall restricts the structure to which structure matching tracking intentionalism appeals to resemblance structure. The approach starts with the familiar observation that qualities and states belong to similarity spaces, i.e. abstract spaces whose geometries are determined by the resemblance relations among their elements. The *maximal (similarity) space* of a given quality consists of all and only the qualities that resemble that quality, along with the distribution of resemblance relations over those qualities. For instance, perhaps the maximal space for true blue is the color similarity space. The structure of a similarity space can be understood as the space considered in abstraction from which elements belong to the space—this allows for the possibility of structurally identical (maximal) similarity spaces that feature different qualities or states. Next, we identify the structure of a quality with its location in the structure of its maximal quality space. The same procedure can be used to define the structure of internal physical states. There will be a perfect structural match between a quality and an internal physical state when the quality and the internal physical state have maximal spaces with the same structure and the location of the quality in its maximal quality space corresponds exactly to the location of the internal physical state in its maximal state space.

To carry the approach further, a graded notion of structural match is needed. One such notion identifies degree of structural match with degree of resemblance. This notion presupposes that resemblance can relate not only qualities and states but also structures.^53^ On this notion, an internal physical state structurally matches a quality just to the extent that its structure resembles the quality’s structure. Alternatively, one could define a graded notion of structure matching in more basic terms. When A and B belong to maximal state or quality spaces with distinct structures, we could take structural differences between those spaces to contribute to structural differences between A and B. One option for formalizing this idea would be to define structural match between spaces in terms of their largest sub-spaces that are structurally identical: we could define the degree of structural match between A’s maximal space and B’s maximal space as the average of the proportions of those spaces covered by their largest structurally identical sub-spaces—meaning the smaller the proportion of the maximal spaces that would need to be deleted to yield structural identity between the spaces, the higher degree of structural match.^54^ Or, when A and B belong to maximal spaces with distinct structures, degree of structure matching between their spaces could be defined in terms of resemblance preservation within a minimal margin of error. On this suggestion, two spaces structurally match to some degree if every pair of elements in the one space resembles to an extent that falls within some margin of error of the extent to which the corresponding pair of elements in the other space resembles. The smaller the margin, the closer the structural match between the spaces. This notion of structure matching would allow spaces that are related by, for example, a stretching transformation to structurally match to a non-maximal degree. With a notion of structural match between spaces, we could then define a degree of structural match between their elements: when A and B belong to maximal spaces with the same structure, we could define their degree of structural match as the proximity of their locations in that structure; when A and B have corresponding locations despite belonging to maximal spaces with different structures, we could equate their degree of structural match with the degree of structural match between their spaces; and when A and B belong to structurally different maximal spaces while also occupying disparate locations, we could take their degree of structural match to be a joint product of sorts between the degree of structural match between their spaces and the proximity of each to the corresponding location of the other.

No doubt other definitions of structure and structure matching are available. In the event that resemblance structure is insufficient to pick out the determinates of tracked determinables, there would remain the option of appealing to a richer notion of structure. Such enrichment could be achieved by defining the operative notion of structure in terms of resemblance along with other sources of the problem of structural correlations. Again, these include compositional relations, magnitudes, determinate-determinable relations, and exclusion relations.

We have sketched options for precisifying structure and structure matching as a proof of concept that structure matching tracking intentionalism admits of development along these dimensions. While we take exploring these and other options to be a promising research direction for those interested in the prospects of tracking theories, undertaking such investigation exceeds the scope of this paper. In what follows, we will set to the one side the question of how best to precisify structure matching tracking intentionalism and continue working with intuitive notions of structure and structure matching that admit of different precisifications. (Anyone who finds these notions objectionably imprecise is invited to read the discussion in terms of one of the suggested precisifications or substitute their own.)

*Second*, internal structure is structure of the state which is determined by the intrinsic physical features of the subject. This allows that an internal state may have structure in virtue of its relations to other internal states and, in turn, that such structure may play a role in delivering a determinate. But structure matching tracking intentionalism is neutral on whether the relevant structure is holistic in this way or instead intrinsic to states.^55^

*Third*, structure matching tracking intentionalism is silent on which of neural, cognitive, and behavioral structure are relevant to delivering a determinate.^56^ Versions of the theory may vary in plausibility depending on which feature(s) they take to be relevant. For example, the grain of phenomenology may be finer than the grain of cognitive and behavioral structure. If so, then versions of the theory that merely appeal to cognitive and behavioral structure will fail to account for fine-grained phenomenology. In contrast, versions that appeal to more fine-grained neural structure may avoid this problem.

*Fourth*, different versions of structure matching tracking intentionalism will issue different predictions about which determinables are tracked and which determinates are further selected. For instance, the determinable tracked for experiences as of true blue might be the determinable blue or the determinable having color. Versions of the theory may vary in plausibility by which determinates they deliver as tracked in a given case, just as simple tracking theories vary in plausibility by which features they deliver as tracked in a given case. In addition, it may be that the best candidate for identification with tracking on simple tracking theories is not the best candidate on structure matching tracking intentionalism. Indeed, since simple tracking theories and structure matching tracking intentionalism accord distinct (albeit similar) roles to tracking, it would be unsurprising for a given candidate identification to vary in plausibility on the two theories. That said, the differences between simple tracking theories and structure matching tracking intentionalism do not immediately suggest that the latter will require an altogether different kind of relation to play the tracking role: given that we sometimes experience determinables but not determinates (e.g. in the visual periphery), even simple tracking theories must allow that determinables can be tracked.^57^

*Fifth*, just as simple tracking theories can be combined with different views of sensory qualities, so too can structure matching tracking intentionalism. Reductive physicalist, primitivist, and quiddity-involving accounts of sensory qualities remain on the table. As far as we can tell, these accounts retain essentially the same costs and benefits in the move from simple tracking theories to structure matching tracking intentionalism. For instance, reductive physicalist accounts remain more parsimonious but are subject to the worry that they cannot account for color structure. On the other hand, primitivist and quiddity-involving accounts require additional ontological commitments but hold more promise when it comes to accounting for color structure.

*Sixth*, on structure matching tracking intentionalism, internal and external factors both substantively constrain phenomenology. Hence, it is a two-factor theory. In contrast, while simple tracking theories place an internal requirement on having an experience—namely that of being in an internal physical state that occupies the e-role—they do not assign internal features a role in substantively constraining the character of experience and so fail to qualify as two-factor theories. While two-factor views of mental content have been discussed in the literature, there has been relatively little explicit discussion of two-factor accounts of what fixes phenomenology.^58^ A notable and relevant exception is Pautz (2006, pp. 232–233), as he raises two objections to two-factor forms of intentionalism that might seem to tell against structure matching tracking intentionalism.

His first objection is that two-factor externalist intentionalism delivers the wrong verdicts in (what we have been calling) SIDE cases. The second objection is that, while two-factor theories of belief enjoy intuitive support, two-factor theories of what fixes phenomenal character enjoy neither intuitive nor theoretical support. Our reply is that, as will be shown in Sects. 5, 6, structure matching tracking intentionalism enjoys theoretical support as it succeeds where rival theories fail in respecting motivated constraints, along with the correct verdicts in SIDE and DISE cases.

In addition, Pautz (2011, pp. 412, 413) considers a disjunctivist two-factor theory in the context of a Mabel-Maxwell Scenario. He rejects it because it requires but fails to provide an explanation of widespread perceptual success. Structure matching tracking intentionalism is immune to this objection as it does not require such success. Indeed, on structure matching tracking intentionalism, even in optimal conditions nothing constrains internal structure to select a determinate for presentation in experience that matches a determinate in the environment. Thus, on structure matching tracking intentionalism one would expect widespread misrepresentation of determinates—at least given that determinate qualities such as true blue and greenish-blue exclude one another. We claim that this is a feature rather than a bug of the theory: whereas simple tracking theories struggle to accommodate such misrepresentation, structure matching tracking intentionalism offers a straightforward account of it.^59^

*Seventh*, as formulated, structure matching tracking intentionalism only allows an internal physical state to select a determinate of a tracked determinable for presentation in experience if there is a *unique* determinate of that determinable whose structure most closely matches that of the internal physical state’s structure. Thus, the theory does not allow internal physical states to select any determinate for presentation in experience in cases where more than one determinate of the tracked determinable has a structure that most closely matches the internal physical state’s structure. This is not obviously the correct verdict for all such tie cases. Thus, structure matching tracking intentionalism is at risk of delivering the incorrect verdicts in these cases.

To construct such a tie case, we might posit a symmetric quality space, suppose that a determinable corresponding to a symmetric region of that space is tracked, and stipulate that the subject is in an internal physical state whose structure most closely matches that of two structurally identical determinates of that determinable. On reflection, however, it is not obvious that any sensory qualities in our world’s nomic neighborhood belong to a symmetric quality space.^60^ More generally, it is not obvious that tie cases of the sort that would be a problem for structure matching tracking intentionalism are nomically possible. Even so, it should be acknowledged that such cases may be possible and, if so, that the theory may need to be tweaked to handle them. A range of modifications is available: the theory could be tweaked to say that ties (for closest structural match) result in an experience as of the tracked determinable but none of its determinates, as of all the determinates of the tracked determinable that themselves have as determinates the tied determinates, or as of all of the tied determinates.^61^ Or the theory could be tweaked to say that ties result in the subject having district experience as of each of the tied determinates, randomly having just one of those experiences, or in being in a state that is indeterminate between all of those experiences. Some of these options may go with some theoretical packages better than others. For instance, reductive views of experience may lend to the indeterminate state option,^62^ while the random state option may fit better with a dualist view that takes psychophysical laws to be fundamental.^63^ We leave it as an open question what the best structure matching tracking intentionalist treatment of tie cases is.

## Problems solved and constraints respected

We are now in a position to see how structure matching tracking intentionalism gives the correct verdict in both the Mabel-Maxwell SIDE and DISE cases. Without loss of generality, we will focus on the color versions of these cases.

In the DISE case, Mabel and Binary Maxwell exhibit internal structural variation: Mabel undergoes N_U_ and exhibits unitary color cognition and behavior while Binary Maxwell undergoes N_B_ and exhibits binary color cognition and behavior. Despite these differences, both of those neural states track some property P of the orange chip. As we saw, simple tracking theories therefore (incorrectly) predicted that Mabel and Binary Maxwell have the same phenomenology. In contrast, structure matching tracking intentionalism does not generate that prediction. Instead, it predicts that Mabel and Binary Maxwell will have structurally different phenomenology that reflects their accompanying internal structural differences. So, if N_U_ and N_B_ exhibit unitary and binary structure, so too will Mabel’s and Maxwell’s phenomenology. Thus, structure matching tracking intentionalism readily avoids the sorts of structural divergence wrought by DISE cases that tells against simple tracking theories.

In the SIDE case, Mabel and Unitary Maxwell are each in the neural state N_U_. Each subject exhibits unitary color cognition and behavior. But, in Unitary Maxwell, N_U_ underlies a unitary color experience and tracks P*, a quality of the red paint chip. In contrast, in Mabel, N_U_ tracks P, the property that N_B_ tracks in Binary Maxwell when he has a binary color experience. As we saw, simple tracking theories therefore (incorrectly) predicted that Mabel has a binary color experience, and hence one that differs in structure from Unitary Maxwell’s. Structure matching tracking intentionalism avoids this result and instead predicts that internal structural similarity engenders structural similarity in phenomenology.

In fact, on structure matching tracking intentionalism, Mabel and Unitary Maxwell may have the same phenomenology. To see this, suppose that in Maxwell N_U_ and N_B_ each track the determinable property of having color. By the stipulations of the case, in Mabel N_U_ tracks the property tracked by N_B_ in Maxwell. Hence, in Mabel, N_U_ also tracks the property of having color. On structure matching tracking intentionalism, the structure of Mabel’s and Unitary Maxwell’s states then settles which determinate of that determinable is presented in their experiences. Our view can readily deliver the correct prediction by noting that the states in Mabel and Unitary Maxwell have the same relevant internal structure.

Unsurprisingly, in addition to delivering the correct verdicts about SIDE and DISE cases, structure matching tracking intentionalism also respects the two constraints we gleaned from those cases. It respects Internal Structural Sensitivity by according internal structure a role in fixing phenomenal structure. And it respects External Structural Insensitivity by precluding mere external structural differences between states that track the same determinable from inducing any structural phenomenal differences—this is how the theory handles cases like that of Mabel and Unitary Maxwell. Moreover, like simple tracking theories, structure matching tracking intentionalism also respects Relationality, Intentionality, and Externalism by assigning tracking a role in fixing phenomenology.

Finally, structure matching tracking intentionalism offers an elegant solution to the problem of structural correlations. The problem was that of accounting for systematic internal-phenomenal structural correlations. This problem is difficult for simple tracking theories because they do not posit (or naturally lend to) any explanatory link between such systematically correlated features. In contrast, structure matching tracking intentionalism tethers phenomenal structure to internal structure. Thus, on structure matching tracking intentionalism, the solution to the problem of structural correlations is that internal structure helps fix phenomenal structure.

## A further advantage: missing shade cases

We have introduced structure matching tracking intentionalism and explained how it succeeds where other theories fail in respecting five motivated constraints and solving the problem of structural correlations. The theory’s appeal does not end here. Below we will show how structure matching tracking intentionalism is unthreatened by an additional problem that afflicts simple tracking theories. This problem concerns what we’ll call *missing shade cases*. In missing shade cases, a subject experiences a quality that, due to its absence from the relevant part of the environment, is not tracked by any of her states. A variety of evidence suggests that missing shade cases are nomically possible if not actual. It is unlikely that simple tracking theories will be able to give a unified explanation of this evidence.^64^ In contrast, structure matching tracking intentionalism can do so.^65^

Some evidence concerns color experience. Experiments indicate that locking certain images on the retina induces experiences of “impossible colors”: reddish-greens and bluish-yellows.^66^ Plausibly, given that nothing can have these colors, they are not tracked by states of subjects who experience them. There is evidence that a recently created substance exhibits a non-naturally occurring blue color.^67^ If so, at least on simple tracking theories that construe tracking as a historical relation, subjects who experienced the color around the time that the substance was created were not in states that track the color. Human “tetrachromats” also pose problems for these theories: some individuals possess genes for two types of red cone and as a result develop neural architecture that allows them to make more fine-grained color distinctions—and, plausibly, have a wider range of color experience—than individuals with just three types of cone.^68^ It is doubtful that the states underlying the distinctive color experiences of such tetrachromats were selected for in humans as a result of their covarying with the colors presented by those experiences.

Other evidence concerns non-visual experience. For instance, there is evidence that by stimulating the bones around the ear, human subjects can experience sounds up to 100 kHz, well outside the range of normal human hearing of roughly 20 Hz to 20 kHz.^69^ There are also many flavors and smells that provide similar cases, including scents of synthetic substances like gasoline as well as characteristic flavors of foods that became widely available only relatively recently, such as bananas, chocolate, coffee, corn, maple, papayas, peanuts, pineapples, tomatoes, and truffles. Again, at least on many historical accounts of tracking, human subjects are not in states that track these qualities. For simple tracking theories on which tracking is a forward-looking relation, there are many cultivars of foods that are no longer grown and of which no seeds are known to exist, and in the near future other foods will likely follow suit (e.g. honey).

Even in the highly unlikely event that none of these are actual missing shade cases, there will presumably be nearby nomically possible missing shade cases. Once the nomic possibility of such cases is admitted, simple tracking theorists could try to treat them in a piecemeal fashion. For instance, these theorists might construe tracking as a disjunctive relation that is defined in terms of other candidate tracking relations. They might incorporate a disjunct that handles the cases that told against historical accounts of tracking with a forward-looking relation, and a disjunct that handles the cases that told against forward-looking accounts of tracking with a historical relation, and another disjunct to give a compositional treatment of cases involving impossible qualities.^70^ Perhaps the resulting theory would be extensionally adequate. But its explanation of missing shade cases would be inelegant. A unified framework for dealing with such cases would be theoretically preferable. To find such a framework one need look no farther than structure matching tracking intentionalism: on it, a subject can experience a missing shade of blue by entering a state that both tracks (say) blueness and suitably matches that missing shade in structure.^71^

## Footnotes

1 See Neander (2017), Shea (2018), Millikan (1984, 2013). N.B. these psychosemantic theories are not theories of experience.

2 We use ‘state’ in a relatively neutral way that is compatible with states being events or processes.

3 There are even naturalistic forms of *naive realism*, the view that, at least in veridical cases, perceived external features constitute phenomenal character. In practice, naive realists typically do not situate their views within the naturalization program. For this reason, we will mostly set aside naive realism. However, simple naive realist theories exhibit some of the same virtues and vices as the simple tracking theories we discuss below. And the theory we propose could be conjoined with naive realism to yield a more sophisticated naive realist theory that is free from some of those vices.

4 Cf. Chalmers (1996).

5 Here and throughout we assume the modest realist view of sensory qualities according to which objects sometimes have some of the sensory qualities that we experience them as having. We claim only that this form of realism is a reasonable starting point, not that it is obviously correct or beyond question.

6 In formulating theories, we use ‘*A* iff *B*’ as short for ‘of nomic necessity, *A* just in case and because *B*’. It follows from this stipulation that *A* iff *B* entails that *B* fixes *A*.

7 Those who prefer to think of what is represented as propositions should feel free to understand the discussion in terms of propositions concerning sensory qualities rather than sensory qualities themselves.

8 See Davidson (1987).

9 Herein lies a key difference between Swampy and the original Swampman: while Swampman lacks historical connections to sensory qualities, his embodiment affords him forward-looking physical connections to them. Not so with Swampy: evidently, it lacks significant discriminating physical connections with sensory qualities generally.

10 See Block (2019, pp. 221–226), Papineau (2021), Pautz (2014a, pp. 155-159; 2019, pp. 390-391), Saad (2021, §3), and Tye (2007a).

11 Such internalists need not deny that, in ordinary circumstances, sensory experiences represent features of their environment. See, e.g., Papineau (2021, p. 115).

12 Tracking theory defenders, proponents, and sympathizers include see Armstrong (1999, Ch. 10, 12), Byrne & Tye (2006), Cutter & Tye (2011), Dretske (1995), Hill (2009, p. 75), Hilbert & Klein (2014), Kriegel (2011), Lycan (1996, 2001), Saad (2021), and Tye (1995, 2000); cf. Kraut (1982). For an overview of tracking theories, see Bourget & Mendelovici (2014). To be clear, some offer simple tracking theories as programmatic suggestions rather than as candidates for true theories—see Byrne & Tye (2006). On such an understanding, one can see the discussion below as developing these suggestions.

13 Thus, playing the e-role stands to experience in roughly the way that, on representational theories of mind, playing the belief-role (i.e. occupying the functional role characteristic of belief) stands to belief.

14 Lycan (1996, pp. 134–135) allows that non-representational features may do more than fix whether one undergoes experience and hence is not strictly speaking a simple tracking theorist. For argument that Lycan's view has problems with scenarios like the one wielded against simple tracking theories in §3, see Pautz (2014b, pp. 294-295).

15 See Cutter & Tye (2011) and Tye (1995, p. 223, note 6); cf. Stampe (1977). Compare Nozick’s (1981) appeal to a covariation relation in his tracking theory of knowledge.

16 See Dretske (1995, pp. 48–49).

17 See Armstrong (1999, p. 143), Millikan (1984, p. 93), Nanay (2013), and Papineau (1984).

18 If perception is a mental relation, then naturalists who invoke it in an account of experience will also need to explain perception in non-mental terms. To find the materials for such an account, such theorists might look to recent work on unconscious perception—see Phillips (2018). Alternatively, such theorists might account for perception in terms of one of the candidate response relations listed above. If they account for perception in terms of, say, co-variation, then the resulting perceptual tracking theory would be a version of, rather than a rival to, covariational tracking theories.

19 See Fodor (1987, p. 108) and Tye (2000, p. 140).

20 See Dretske (1995, Ch. 1); cf. Tye (2000, Ch. 6, §6.1).

21 See Nanay (2014).

22 See Block (1998).

23 See Tye (1995, p. 101, 2000, Ch. 6, §6.4).

24 See Mehta & Mashour (2013, §2) for an overview.

25 See Byrne & Hilbert (2003).

26 See Campbell (1993).

27 See Cutter (2018).

28 Tracking theories fit better with a response-independent view of sensory qualities for several reasons. First, the most phenomenologically plausible response-dependent views of sensory qualities construe them as dispositions to cause certain experiences (see, for example, Johnston (1992)). Combining such a view with a tracking theory risks circularity. To illustrate, suppose that redness = the property of being disposed to cause a certain sort of experience. If a tracking theory is correct, what it takes to cause that sort of experience presumably depends on what it takes to track redness; but if redness is identical with the noted dispositional property, then what it takes to track that property also depends on what it takes to cause that sort of experience. Second, response-dependent properties seem like poor candidates for the states that our brain states track—this is perhaps especially so on response-dependent views that identify sensory qualities with dispositions to produce certain neural responses (Pautz, 2010b, §5). Third, it is extremely plausible that spatial sensory qualities (e.g. roundness) are response-independent. However, once a response-independent view of spatial sensory qualities is accepted, there is pressure to extend the response-independent view to other sensory qualities such as colors. In addition to uniformity considerations, there is also the intuition that, necessarily, whatever has color has shape. This intuition tells against the hypothesis that spatial sensory qualities are response-independent while color sensory qualities are response-dependent: for if color are response-dependent while shapes are response-independent, then a shapeless point-particle could have color by being disposed to produce the relevant response (Cutter, 2016). Those who are more optimistic about combining tracking theories with response-dependent views of sensory qualities are invited to consider the consequences of substituting their preferred response-dependent account into our discussion of tracking theories.

29 Unless otherwise indicated, ‘states’ should be read as referring to internal physical states that occupy the e-role.

30 E.g., see Hurley (1998, p. 253), Papineau (2014, p. 17), and Strawson (2008, p. 152), as well as those cited in Pautz (2014a). Cf. Lewis (1999, pp. 315–316).

31 See Dretske (1995, p. 145) and Tye (2000, p. 64).

32 Cf. Pautz (2014a, *passim*).

33 For these scenarios, respectively see Davidson (1987); Harman (1982) and Block (1990); Tye (2000, Ch. 6); Russell (1921: Lect. IX); Hurley (1998, pp. 314-323) and Thompson (2010, §7); Chalmers (2012, p. 334); Thompson (2010, §3); Pautz (2010a); Pautz (2017, pp. 381–386); unpublished lectures by Kripke; and Hume (1738-40: Part 1, Book 1) and Neander (2017, pp. 200-204).

34 It may not be immediately obvious that each of these scenarios poses a problem with this form. For instance, consider Swampman. Since Swampman lacks a history, he does not track anything on views that construe tracking as a historical relation. Thus, trivially, on such views Swampman’s internal structure is dissociated from tracked structure.

35 While Pautz generally uses the Scenario as a DISE case, which he accords varying purposes, we use the Scenario to generate both SIDE and DISE cases along with corresponding structural constraints on theorizing about experience.

36 Pautz (2010a, pp. 33–34; 2010b: 335-40; 2014b: §18.2) also gives analogous scenarios involving other sorts of experience, along with accompanying evidence of phenomenal-neural structural matches in actual humans.

37 Here we use color terms to denote sensory qualities while remaining neutral on whether ordinary uses of color terms do so as well.

38 Why not stipulate that the subjects track orange and red, rather than P and P*? One reason is to allow tracked features to encompass structural features that are presented in color experience, rather than just the experienced colors. Another reason is that, as we will see in §4, we should allow that the tracked features are distinct from but systematically related to the experienced qualities.

39 A salient option is to account for the phenomenal binary-unitary distinction in terms of whether (and if so how) a given color is experienced as having other colors as components. Alternatively, the distinction might be explained in terms of features of phenomenal color space such as metrical structure, dimensionality, or directionality—cf. Clark (1993, pp.126-130).

40 For an attempt to recover such structure using resources available to standard physicalists, see Byrne & Hilbert (2003, p. 13) and Tye (1995, pp. 148–149; 2000, pp. 162–165). For non-reductive treatments of color, see Allen (2015) and Cutter (2018).

41 For instance, maybe N_U_ was selected for in Mabel’s species because it causally covaries with P.

42 Simple tracking theorists who opt for a radical form of interactionist dualism may take this stipulation to render the Scenario nomically impossible. For they may hold that the differences in what features are tracked by Mabel and Unitary Maxwell lead to phenomenal differences that induce behavioral and cognitive differences—see Saad (2019a, 2019b). Simple tracking theorists who opt for physicalism or milder forms of dualism cannot avail themselves of this response, as the stipulated physical similarities between the two subjects leave such theorists without a basis for predicting a divergence in cognitive and behavioral structure. We bracket the noted form of dualism in what follows.

43 See Logvinenko & Beattie (2011) and Logvinenko & Geithner (2015). Interestingly, however, some experiments have not found behavioral markers of the unitary-binary distinction that one might have expected—e.g., see Forder et al., (2017, p. 5).

44 See, for instance, Brouwer & Heeger (2009, 2013), Forder et al. (2017), Parkes (2009), and Wuergerand & Parkes (2011). Fortunately, these results do not presuppose—and, in some cases, in fact conflict with—the now dubious opponent-process account of the unitary-binary distinction in color experience (Valberg, 2001).

45 The parenthetical restriction encodes a way of running the objection using a weakened assumption. However, we do not see how the objector could exploit the weakening to plausibly resist our below reply. So, for simplicity, we will only answer the non-parenthetical reading of the objection.

46 See Block (1999). Because such subjects generally differ at the receptor level, one could question whether they are responding to the same quality of the stimulus and in turn whether such cases feature the requisite sort of mismatch on the no-interference account of optimal conditions—cf. Pautz (2014b, p. 253). However, even if actual cases of this sort turn out not to qualify as mismatch cases on that account, it is clear that nomically possible cases along these lines will: there could be human-like subjects who are the same at the receptor level, but whose post-receptoral wiring—in the absence of interfering factors—leads them to enter structurally different states in response to true blue.

47 See Byrne & Hilbert (2007, fn6), Pautz (2006, p. 214) and Tye (2006, pp. 175-6; 2007*b*: 93).

48 See Pautz (2014b, pp. 249–251).

49 Here understood as relations of being necessarily, possibly, or not possibly co-instantiated with.

50 Cf. Cutter (2021, p. 400).

51 Neander (2017, ch. 8) also makes use of determinate-determinable relations in her account of content-fixing. Cf. Dretske (1995, p. 2).

52 Cf. Newman (1928).

53 Compare: in some possible world semantics, possible worlds are held to resemble to different degrees (Starr, 2019).

54 Cf. Lee et al. (2022, §4).

55 See Neander (2017) and Shea (2018) for some accounts of structure in the context of psychosemantics that could be co-opted by structure matching tracking intentionalism.

56 It does however require that the structure in question be internal. This will disqualify external cognitive structure and manifest behavioral structure, though it leaves room for internal cognitive structure and the structure of behavioral dispositions.

57 One might worry that this is a problem for simple tracking theories if only maximally determinate properties can cause effects. However, reflection on ordinary cases in which maximally determinate properties feature causally irrelevant detail independently supports the causal efficacy of determinables (Yablo, 1992).

58 Some theorists discuss views that are at least in the vicinity but it's not clear that these views are two-factor theories of phenomenology. In some cases, that's because it's not clear that one of the factors is internal ((Hilbert & Kalderon, 2000, p. 198), (Logue, 2012, pp. 222–223)); in others, because it's not clear that one of the factors is external ((Beck, 2019, pp. 625–626), (Berger, 2018, fn22, p.c.)); and in still others because it's not clear that the views take the two factors to determine phenomenal character rather than something else ((Beck, 2019, pp. 625–626), (Hill, 2009; 2012, fn2), (Lycan, 1996, p. 140)).

59 The problem of accommodating reliable misrepresentation is widely discussed in the psychosemantics literature. For applications to experience, see Mendelovici (2013, 2016). For those who take widespread perceptual success as a datum to be accommodated, there are ways to accommodate it on structure matching tracking intentionalism. One option for securing widespread perceptual success on structure matching tracking intentionalism is to appeal to a pluralist view of sensory qualities on which objects that have determinable sensory qualities also have all their determinates (cf. Kalderon (2007)). Another option is to appeal to a fine-tuning mechanism, such as a designer or axiarchic principle, that biases the distribution of physical states in our world toward inducing a match between the structure of subjects’ internal states and the structure of certain determinates in the environment and, in turn, toward yielding widespread perceptual success.

60 See Byrne (2020: §2) and Hilbert & Kalderon (2000).

61 Cf. Crane & Piantanida (1983).

62 See Papineau (2003).

63 Cf. Bourget (2019).

64 Alternatively, one could try to bring missing shade cases to bear on simple tracking theories in the following way: missing shade cases are open empirical possibilities; but if these theories are true then missing shade cases are nomically impossible; so, the fortunes of simple tracking theories are tethered to an open empirical issue (cf. Mendelovici (2016)). However, it would be a mistake to take this to thereby reveal anything problematic about simple tracking theories. Neutrality with respect to open empirical issues is not a theoretical virtue.

65 Neander (2017, pp. 200–203) suggests that it is a strength of her “causally driven analogs and teleosemantics”—a kind of holistic theory of intentionality (but not of experience)—that it handles missing shade cases. However, she does not present evidence for such cases. The evidence provided below could be used to bolster her suggestion.

66 See Billock et al. (2001) and Crane & Piantanida (1983).

67 See Smith et al. (2009).

68 See Jameson (2009).

69 See Qin (2011).

70 A compositionalist treatment for a given composite quality would take the states that track that quality to do so by being a concatenation of states that track components of that quality. To illustrate, simple tracking theorists could give a compositionalist account of experience as of reddish-green by maintaining that the subject is in a state that tracks red, that she is in another state that tracks green, and that these states combine in a manner that yields a composite state that thereby tracks a composite quality, namely reddish-green. However, this does not provide a promising approach to dealing with all missing shade cases. Witness the marked difficulty of describing the scents and flavors above in terms of other sensory qualities.

71 Cf. Dretske (1995, p. 21).
